# Polyarticular juvenile idiopathic arthritis (pJIA): clinical and serologic predictors of inactive disease (ID)

**DOI:** 10.1186/1546-0096-12-S1-P196

**Published:** 2014-09-17

**Authors:** Silvia Meiorin

**Affiliations:** 1 Hospital de Niños Ricardo Gutierrez, Buenos Aires, Argentina

## Introduction

Juvenile idiopathic arthritis (JIA) is the most common chronic rheumatic disease in childhood and the polyarticular subtype is associated with persistent activity and a low rate of remission. The goal of an early and aggressive treatment is to achieve remission preventing irreversible joint destruction and long-term disabilities.

## Objectives

1) To assess disease activity in patients with pJIA “under early therapy with MTX.”

2) To determine predictors of ID in this cohort**.**

## Methods

Clinical charts of 174 pJIA pts (ILAR´01), (1998 -2009) we reviewed. Inclusion criteria:-pJIA pts under “early” MTX–follow up “a minimum of 12mo”. Demographic, clinical, laboratory, and therapeutic variables were analyzed at disease onset, 12mo and last visit. Functional disability(CHAQ), damage (JADI Articular score) and Remission Criteria (Wallace) were properly assesed.

## Results

Ninety eight patients were included , 85 girls, (86.7%) median age at onset 10 years ( IQR :6 -12) ,median disease duration 60 months ( IQR :36 -74 ) and follow-up 48 months (IQR :24,5 -67) ; 34 pts (34.6%) were RF Positive. All patients received MTX; 32 /98 (32.6%) reached ID at amedian follow up time of 12 mo with a CHAQ mean value 0,37 and a JADI mean value 1,3. Nineteen out these 32 pts (60%) sustained remission for at least 37 mo. ID was associated with a less time of disease evolution (p.05), a better function CHAQ (p .05) and less damage (p .04)A higher prevalence but not significantly RF titers (40 vs 25%); seronegative ANA titers (60 vs 53 ) and RX damage (47 vs 32 ) were observed in the group of non responders pts to MTX. On multivariate analysis a less time of disease evolution was the only predictive risk factor associated to inactive disease (β 1,01 p .05).

## Conclusion

In our series , only 32/98 (32.6%) pJIA pts reached ID , this clinical state was sustained in 60% of them longer than 2 years. A less interval between disease onset and Mtx start was the only variable predicting inactive disease.

## Disclosure of interest

None declared.

